# The effects of rotational setup errors in total body irradiation using helical tomotherapy

**DOI:** 10.1002/acm2.13271

**Published:** 2021-05-24

**Authors:** Akira Isobe, Keisuke Usui, Naoya Hara, Keisuke Sasai

**Affiliations:** ^1^ Department of Radiation Oncology Graduate School of Medicine Juntendo University Tokyo Japan; ^2^ Department of Radiological Technology Faculty of Health Science Juntendo University Tokyo Japan; ^3^ Department of Radiology Juntendo University Hospital Tokyo Japan

**Keywords:** acceptable level, delivered dose distribution, helical tomotherapy, rotational setup error, total body irradiation

## Abstract

**Purpose:**

Helical tomotherapy (HT) is a form of intensity‐modulated radiation therapy that is employed in total body irradiation (TBI). Because TBI targets the whole body, accurate setup positioning at the edge of the treatment volume is made difficult by the whole‐body rotational posture. The purpose of this study is to clarify the tolerance for rotational setup error (SE) in the vertical direction. In addition, we perform a retrospective analysis of actually irradiated dose distributions using previous patients’ irradiation data.

**Methods:**

To clarify the effects of rotational SE on the dose distribution, the planned CT images of 10 patients were rotated by 1–5° in the vertical (pitch) direction to create a pseudo‐rotational SE image. Then, the effect of the magnitude of the rotational SE on the dose distribution was simulated. In addition, the irradiated dose to the patients was analyzed by obtaining recalculated dose distributions using megavoltage CT images acquired before treatment.

**Results:**

The simulation results showed that the average value of the lung volume receiving at least 10 Gy did not exceed the allowable value when the SE value was ≤2°. When the rotational SE was ≤3°, it was possible to maintain the clinical target volume dose heterogeneity within ±10% of the prescribed dose, which is acceptable according to the guidelines. A retrospective analysis of previous patients’ irradiation data showed their daily irradiation dose distribution. The dose to the clinical target volume was reduced by up to 3.4% as a result of the residual rotational SE. Although whole‐course retrospective analyses showed a statistically significant increase in high‐dose areas, the increase was only approximately 1.0%.

**Conclusions:**

Dose errors induced by rotational SEs of ≤2° were acceptable in this study.

## 1 INTRODUCTION

Total body irradiation (TBI) combined with chemotherapy is widely used as a pre‐bone marrow transplant regimen in hematological malignancies, and it has superior treatment results to those of chemotherapy‐only regimens.^1^, ^2^, ^3^ Helical tomotherapy (HT) is a form of intensity‐modulated radiation therapy that improves target dose uniformity and reduces the dose to organs at risk. Thus, HT has been used as a safer method of TBI administration.^4^, ^5^, ^6^ Because TBI targets the whole body, the whole‐body rotational posture makes accurate setup positioning at the field edge difficult. Additionally, when irradiating a large target, as in TBI, the patient couch sag peculiar to the HT apparatus increases, generating systematic rotational error in the vertical (pitch) direction.^7^ However, HT systems are not equipped with a function to correct the rotational setup error (SE) in the pitch direction.^8^ Furthermore, surface dose deviations due to SE have major effects in HT radiotherapy.^9^, ^10^ Takenaka et al. recommended that the translational SE in the horizontal direction be within 5 mm in TBI with HT.^10^ However, the dosimetric impact of rotational SE in the pitch direction in TBI using HT is unclear, and reports assessing this disadvantage are not yet available. Repeating the image‐guided radiotherapy (IGRT) process, which means rotational correction in first MVCT and acquire the MVCT again for confirmation position is a problem because it requires a considerably long treatment time and increases patient distress. Moreover, the HT system allows you to skip the scan step, but it is inadvisable to do so because the external skin marks may not be a reliable indicator of the target position. In addition, it is impossible to achieve exactly the same patient position as that in the planning CT data. Therefore, knowing the tolerance level of rotational SE could contribute to rationalizing the IGRT process in HT systems.

In this study, we aimed to clarify the tolerance for rotational SE in the pitch direction in TBI using HT. Furthermore, we demonstrated the effects of residual rotational SE on the whole‐body dose distribution by a retrospective analysis of irradiation data from previous patients.

## 2 METHODS

### 2.A Patients and treatment planning

This study’s subjects were 10 patients who underwent TBI and were enrolled from January to December 2018, as approved by our hospital’s Institutional Review Board (reception number: 18‐034). The patients’ characteristics are listed in Table 1.

**Table 1:** Patient characteristics.

Patient	Age	Sex	Height (cm)	Weight (kg)	Disease
1	50	F	154	49	AML
2	44	M	176	72	B‐ALL
3	48	M	174	55	AML
4	53	M	184	62	PMF
5	52	M	168	63	B‐ALL
6	37	M	179	70	MDS
7	42	M	173	59	MDS
8	47	M	178	60	PMF
9	52	F	151	49	MDS
10	18	F	155	50	B‐ALL

AML, acute myeloid leukemia; B‐ALL, acute B‐lymphoblastic leukemia; PMF, primary myelofibrosis; MDS, myelodysplastic syndromes.

**Fig. 1:**
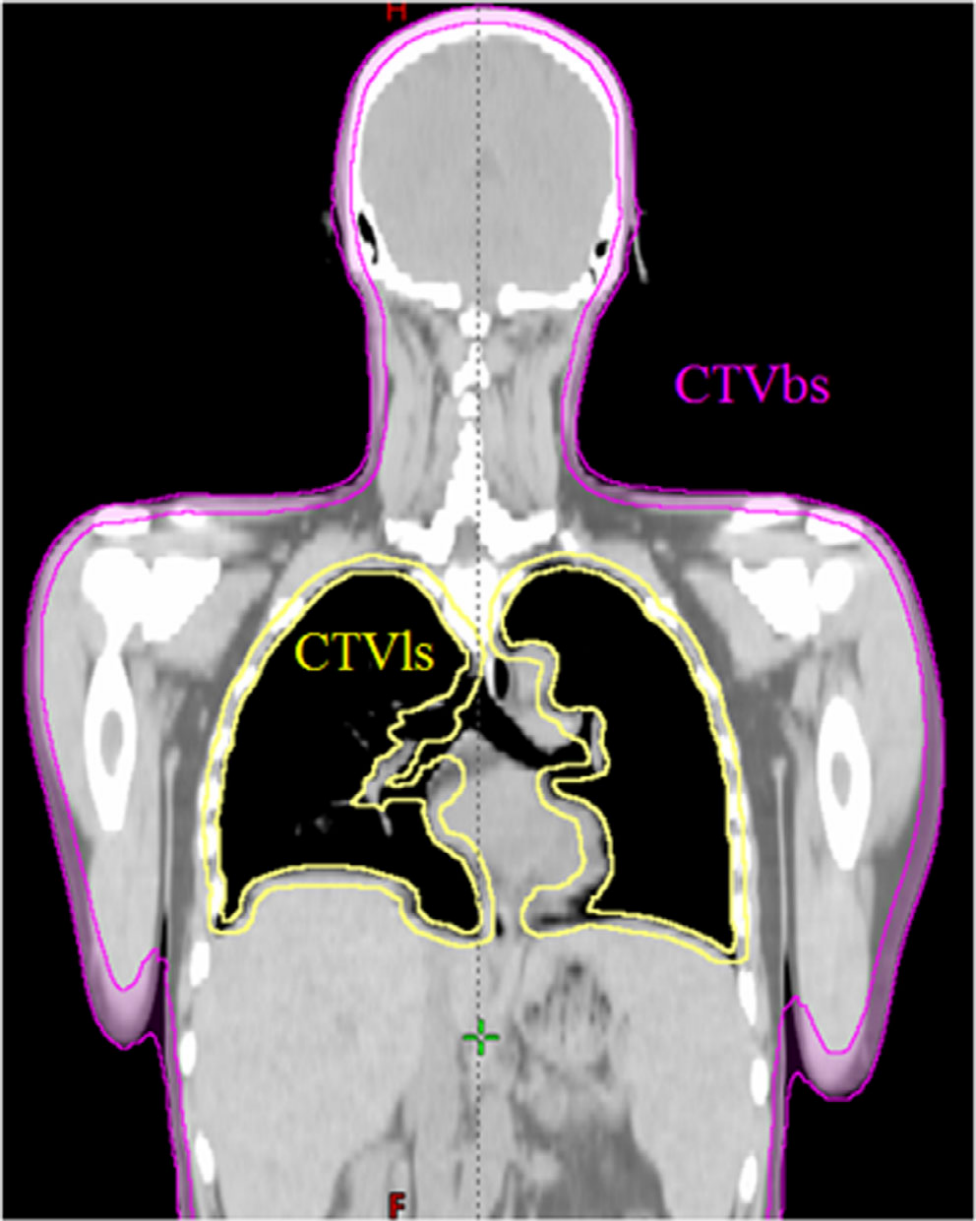
Image example illustrating the definitions of CTVbs (body surface of the clinical target volume) and CTVls (lung surface of CTV). The area within 5 mm of the body surface was defined as CTVbs and shown in pink. The area from the border of the lung to 5 mm outside the lung was defined as CTVls and shown in yellow.

A whole‐body suction fixture and a thermoplastic head mask were used to ensure fixation accuracy. The planning CT data of patients who underwent TBI were obtained. A 5‐mm thick image was obtained using a 16‐sensor data acquisition system‐type whole‐body CT system (Aquilion LB, Canon Medical Systems, Tochigi, Japan). The field of view was 550 mm. Then, separate treatment plans were created for the upper and lower parts of the body. The upper and lower plans were defined as the “head‐first plan” (HF) and the “feet‐first plan” (FF), respectively, according to reversed head‐to‐tail direction with respect to the system. For the safety of the image data acquisition process, the lengths (120 and 100 cm for the upper and lower body, respectively) were controlled to be shorter than the longest irradiation range of the HT system (i.e., 135 cm).^11^, ^12^ The anatomy of the pelvis can be used for image registration between the HF and FF images, which can improve the image registration accuracy. Therefore, the pelvis can be included in both images by acquiring images of length 120 cm and 100 cm from the top of the head and the toes, respectively. Moreover, we need to set the patient up twice for the individual plans (HF and FF plans) while maintaining the patient’s posture. Therefore, we used a Styrofoam (polystyrene) board under the whole‐body suction fixture, and the patient’s position was rotationally exchanged to the other direction on the treatment couch along with the base (Styrofoam board and whole body suction fixture) by many medical staff members. The clinical target volume (CTV) was the whole‐body contour excluding the lung, which is an organ at risk. In consideration of the setup margin, the planning target volume (PTV) was set to a volume obtained by adding 5 mm to the CTV toward the lung contour. This prevented insufficiency of the dose to the sternum and ribs adjacent to the lungs. According to a previous report,^10^ no margin was added to the body contour to prevent an increase in excessively high‐dose areas on the body surface. The prescription dose was optimized by a radiation treatment planning system (TomoHDA System Planning Station version 5.1.1.6, Accuray, Madison, WI, USA) using a constraint to cover 95% of the PTV with 12 Gy. In the FF plan, the CTV was the body contour. In the treatment planning for the HT plan, the field width was 5.0 cm, the pitch was 0.287, and the modulation factor was determined by adjusting each patient’s value from 2.5 as a reference value. To reduce the region of field junctional overdose, we narrowed the target volume by 2.5 cm (5 slices) at both the upper and lower body irradiation junctions.^13^ Moreover, adopting the fixed jaw mode can help to keep the influence of dose difference within the permissible range allowed by the SE. The reason for adopting the fixed jaw mode is that the fixed jaw mode plans can use the natural dose fall‐off beyond the edge of the PTV, which makes it possible to achieve a gradually attenuated dose distribution at the junction. In addition, Two regions were defined as the prediction area of large dose deviations for the CTV in each plan. The outer boundary of the CTVbs (CTV of the body surface) is the contour of the patient's surface. The inner boundary of the CTVbs is expanded 5 mm inward from the patient's surface. The inner boundary of the CTVls (CTV of the lung surface) is the lung contour. The outer boundary of the CTVls is expanded 5 mm outward from the lung contour. (Fig. 1). Additionally, one region was defined as the prediction area of large dose deviations for the CTV in the overall combined plan. The CTVjt region (CTV junction between the HF and FF plans) was defined as that limited to 5 cm (10 slices) in both the head and feet directions from the junction slice in the CTV.

### 2.B Pseudo setup error simulation

To simulate the effects of rotational SE on dose distribution, the planning CT images of each patient were rotated by 1–5° in the pitch direction. In our study, the center in the body axis direction in the CT image was located at the rotational center of the SE. Therefore, the slice number of the CT image at the rotational center was 60 and 50 in the HF and FF plans, respectively. Simultaneously, each organ structure dataset attached to the planning CT image was rotated using commercially available software (MIM Maestro version 6.5.9, MIM Software, Inc., Cleveland, OH, USA). Subsequently, we recalculated the dose distribution for each rotational SE image under the same calculation conditions and using the treatment plans mentioned in Section 2.A. The dose indexes D_98%_ (the minimum absorbed dose that covers 98% of the volume), D_95%_, and D_2%_ were compared for quantitative evaluation of CTV coverage for all dose distributions. D_98%_ and D_2%_ were defined as the near minimum dose and near maximum dose, respectively. Furthermore, D_98%_ and D_2%_ of CTVbs on the body surface and CTVls on the surface in contact with the lung were evaluated. In accordance with the American College of Radiology and American Society for Radiation Oncology guidelines,^14^ V_110%_ (the volume receiving at least 110% of the prescription dose within the CTV) and V_90%_ were used as indicators of high‐ and low‐dose regions, respectively. Dose heterogeneity needs to be maintained within ± 10% of the prescription dose: V_110%_ must be 10% or less and V_90%_ must be 90% or more of the CTV. We quantitatively compared the change in high‐ and low‐dose regions within the CTVs between the original and simulation doses. The lungs were evaluated for mean dose and V_10Gy_ (the volume receiving at least 10 Gy). The V_10Gy_ value was not allowed to exceed 40% of the whole lung volume, with reference to the predictors of radiation pneumonia in patients who underwent radiation therapy.^15^ Therefore, changes in the dose distribution within the CTVs and lungs due to increased rotational SE were compared with the original plan dose distribution to establish SE tolerance.

Additionally, because of the couch sag and the differences in the patient’s posture, the pitch error exerts its effects in opposite directions in the HF and FF plans. Therefore, we analyzed the impact on the junction area in the overall combined plan. Assuming the pitch offsets in the direction in which the couch is expected to sag situation, we analyzed the effects of rotational SE on the junction area in the head‐down and toe‐down directions for the HF and FF plans, respectively.

The differences between the means of the original planned dose distribution and simulation dose distribution with rotational SE were considered statistically significant when p < 0.05 (two‐tailed t‐test). R version 1.41 (www.r‐project.org) was used for the paired t‐tests.

### 2.C Evaluation of delivered dose distribution

**Fig. 2:**
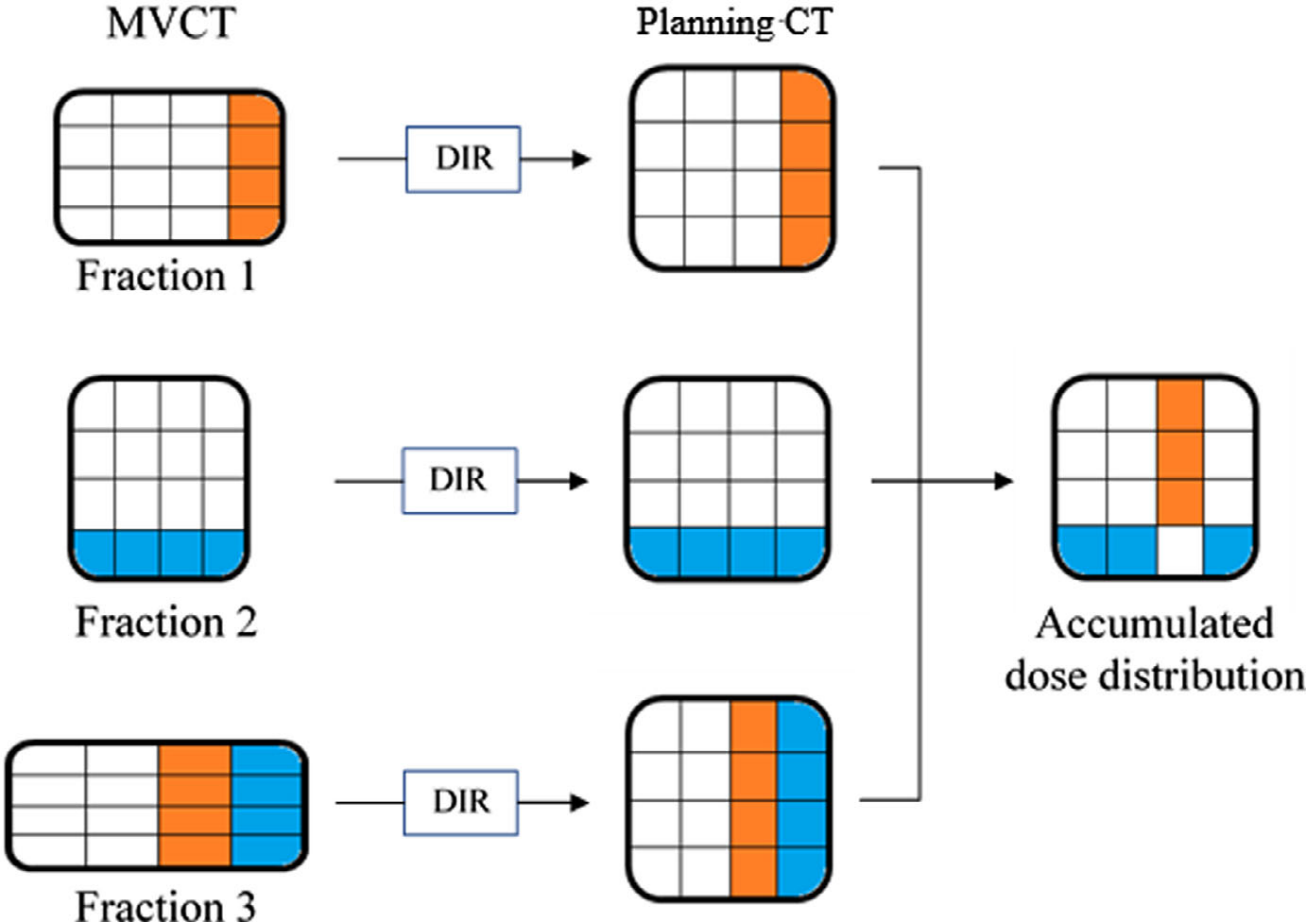
Conceptual diagram from deformation processing of megavoltage CT (MVCT) to the integration process into the planning CT image. Red and blue pixels on the MVCT image indicate 110% and 90% of the prescribed dose, respectively, and thus denote high‐ and low‐dose points, respectively. Because each pixel on the MVCT image is assigned to the corresponding pixel on the planning CT image by deformable image registration (DIR) processing, the dose associated with the MVCT pixels was also assigned to the planning CT pixels’ positions. In the process of integration, high‐dose and low‐dose points may cancel or add to each other. In this figure, the positions of the canceled doses are shown in white, which means no dose difference.

For the HT treatment procedure, we obtained the megavoltage CT (MVCT) data from all treatment periods before each irradiation. Furthermore, the SE correction made using these data was finalized by the radiation therapist after automatic matching by the equipped software (TomoHDA System Operator Station version 5.1.4.3, Accuray) in the HT unit. The detected patient SE was automatically corrected in translation by the HT couch.^16^ Subsequently, the residual of the vertical rotational SE, which was not amendable at the time of the patient irradiation, was detected using software (TomoHDA System Planned Adaptive version 5.1.0.6, Accuray). Moreover, the actual delivered dose to the patient was obtained by recalculation of the dose distribution (TomoHDA System Planned Adaptive version 5.1.0.6, Accuray) using the planned sonograms.^17^ To evaluate the difference between the accumulated dose distribution of each delivered dose and the dose distribution at the time of treatment planning, the obtained MVCT was subjected to deformable image registration (DIR) processing to the planning CT image. The delivered doses associated with the MVCT images were also modified. Hybrid DIR software (RayStation version 6,2.0.7, RaySearch Laboratories, Stockholm, Sweden) that performs deformation using both intensity and anatomical information was used as the DIR processing algorithm.^18^ Figure 2 shows the process of integrating the deformed MVCT images into the planning CT image for comparison of the actual delivered dose. The irradiated dose was assigned to each pixel of the MVCT image by a recalculation process. Then, the position of each MVCT pixel was associated with the corresponding pixel position of the planning CT image by DIR processing. By integrating the doses assigned to the MVCT image pixels throughout the whole course (six fractions) into the pixels of the corresponding planning CT image, we compared the planning dose distribution and irradiated dose distribution with the planning CT image.

## 3 RESULTS

### 3.A Tolerance levels of SE in the pitch direction

Tables 2 and 3 show the dose indexes with various rotational SE values using the HF and FF plans, respectively. In the following text, statistically significant differences are described as significant differences. The mean D_95%_ values ± standard deviation (SD) of the original HF and FF plans in the CTV were 12.0 Gy and 11.9 Gy, respectively, with SD values of <0.1.

**Table 2:** Effects caused by rotational setup error in the pitch direction using the head‐first plan.

	Plan		Rotational SE (degrees) in pitch direction. (Average ± SD)				
			1.0	2.0	3.0	4.0	5.0
CTV	D_98%_ (Gy)	11.6 ± 0.0	11.6 ± 0.0	11.4 ± 0.1*	11.1 ± 0.1*	10.7 ± 0.1*	10.0 ± 0.2*
	D_95%_ (Gy)	12.0 ± 0.0	11.9 ± 0.0*	11.9 ± 0.0*	11.8 ± 0.0*	11.6 ± 0.0*	11.4 ± 0.0*
	D_2%_ (Gy)	12.9 ± 0.0	13.5 ± 0.0*	14.4 ± 0.1*	15.0 ± 0.1*	15.2 ± 0.1*	15.3 ± 0.1*
	V_110%_ (%)	0.6 ± 0.1	2.9 ± 0.4*	5.1 ± 1.0*	7.6 ± 3.4*	10.2 ± 6.8*	12.2 ± 10.7*
	V_90%_ (%)	99.3 ± 0.1	99.4 ± 0.1	99.1 ± 0.2	98.6 ± 0.2*	97.8 ± 0.3*	96.7 ± 0.4*
Lung	Mean (Gy)	9.4 ± 0.0	9.5 ± 0.0*	9.6 ± 0.1*	9.6 ± 0.0*	9.7 ± 0.0*	9.8 ± 0.0*
	V_10 Gy_ (%)	39.1 ± 2.2	38.9 ± 2.5	39.2 ± 2.0	40.6 ± 3.4*	42.8 ± 6.5*	45.3 ± 10.5*
CTVbs	D_98%_ (Gy)	11.0 ± 0.2	10.7 ± 0.1*	10.4 ± 0.1*	9.9 ± 0.1*	9.3 ± 0.2*	8.4 ± 0.2*
	D_2%_ (Gy)	13.2 ± 0.0	14.9 ± 0.2*	15.8 ± 0.1*	16.0 ± 0.1*	15.9 ± 0.1*	15.7 ± 0.1*
CTVls	D_98%_ (Gy)	11.1 ± 0.0	10.7 ± 0.1*	9.9 ± 0.2*	9.0 ± 0.3*	8.2 ± 0.3*	7.5 ± 0.2*
	D_2%_ (Gy)	12.8 ± 0.0	12.8 ± 0.0	12.8 ± 0.0	12.8 ± 0.0*	12.8 ± 0.0*	12.8 ± 0.0*

* *P* < 0.05.

**Table 3:** Effects caused by rotational setup error in the pitch direction using the feet‐first plan.

	Plan		Rotational SE (degrees) in pitch direction. (Average ± SD)				
			1.0	2.0	3.0	4.0	5.0
CTV	D_98%_ (Gy)	11.8 ± 0.0	11.6 ± 0.0*	11.5 ± 0.0*	11.4 ± 0.0*	11.2 ± 0.0*	10.9 ± 0.0*
	D_95%_ (Gy)	11.9 ± 0.0	11.8 ± 0.0*	11.7 ± 0.0*	11.7 ± 0.0*	11.6 ± 0.0*	11.5 ± 0.0*
	D_2%_ (Gy)	12.6 ± 0.0	13.2 ± 0.1*	14.1 ± 0.3*	14.9 ± 0.4*	15.3 ± 0.6*	15.7 ± 0.7*
	V_110%_ (%)	0.1 ± 0.0	1.8 ± 0.8*	5.3 ± 2.4*	7.9 ± 3.2*	10.1 ± 4.6*	11.7 ± 6.0*
	V_90%_ (%)	100.0 ± 0.0	99.9 ± 0.2*	99.6 ± 0.1*	99.4 ± 0.1*	99.0 ± 0.1*	98.3 ± 0.1*
CTVbs	D_98%_ (Gy)	11.6 ± 0.0	11.1 ± 0.0*	10.8 ± 0.1*	10.7 ± 0.0*	10.3 ± 0.0*	9.6 ± 0.2*
	D_2%_ (Gy)	12.7 ± 0.0	13.8 ± 0.2*	15.0 ± 0.5*	15.6 ± 0.6*	16.0 ± 0.8*	16.1 ± 0.9*

* *P* < 0.05.

**Fig. 3:**
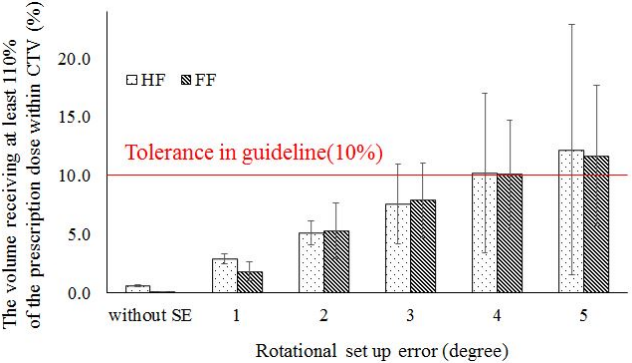
Changes in high‐dose regions of clinical target volume (CTV) caused by rotational setup error. The high‐dose region was defined as V_110%_ (the volume receiving at least 110% of the prescription dose within the CTV). HF and FF mean head‐first plan and feet‐first plan, respectively. Error bars represent the standard deviation in a whole patient.

**Fig. 4:**
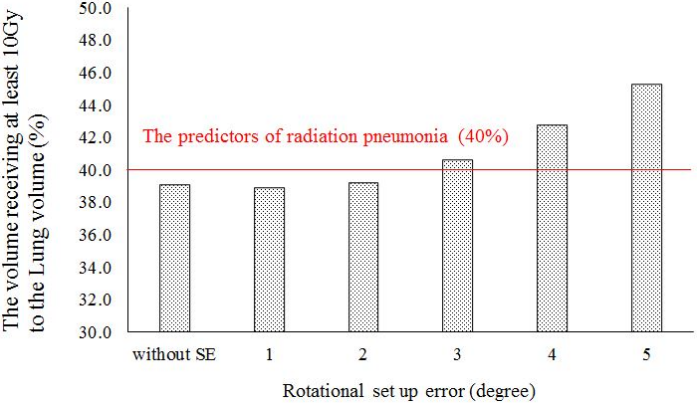
Results of lung V_10Gy_ (the volume receiving at least 10 Gy) in rotational setup error simulations. The V_10Gy_ value was not allowed to exceed 40% of the total lung volume, with reference to the predictors of radiation pneumonia in patients who underwent radiation therapy.

In the HF plan, the D_95%_ and D_98%_ values in the CTV decreased with increasing SE. A statistically significant difference in D_98%_ was found between the original planned dose and the delivered dose when the SE was ≥2°, and there was a significant difference in D_95%_ at SE values of ≥1°. The CTV D_2%_ value increased with increasing SE, with a statistically significant difference from the original planned dose shown for SE values of ≥1°. The CTV V_110%_ value increased with increasing SE, with a statistically significant difference from the original planned dose shown for SE values of ≥1°. Figure 3 shows the changes in the high‐dose regions (V_110%_) of the CTV owing to rotational SE. When SE was ≥4°, the average CTV V_110%_ value exceeded 10%, which is the guideline tolerance. The CTV V_90%_ value decreased with increasing SE, with a statistically significant difference from the original planned dose shown for SE values of ≥3°. The mean lung dose was significantly different from the original planned dose when the SE value was ≥1°, with a gradual increase shown with increasing SE. The lung V_10Gy_ value showed a statistically significant difference from the original planned dose at SE values of ≥3°. The V_10Gy_ value in the lung decreased slightly at the SE value of 1° and increased at ≥2°. Furthermore, at SE values of ≥3°, the mean lung V_10Gy_ value exceeded the allowable value of 40%. Figure 4 shows the results of lung V_10Gy_ simulation. The CTVbs D_98%_ value decreased with increasing SE. The CTVbs D_2%_ value increased to a maximum at 3° with increasing SE, but the value subsequently decreased at SE values of ≥4°. Both CTVbs D_98%_ and D_2%_ were significantly different from the original planned dose at SE values of ≥1°. The CTVls D_98%_ value decreased with increasing SE, and there was a statistically significant difference from the original planned dose at SE values of ≥1°. The CTVls D_2%_ value showed no statistically significant differences between the original planned and simulated doses at SE values of ≤2°.

**Fig. 5:**
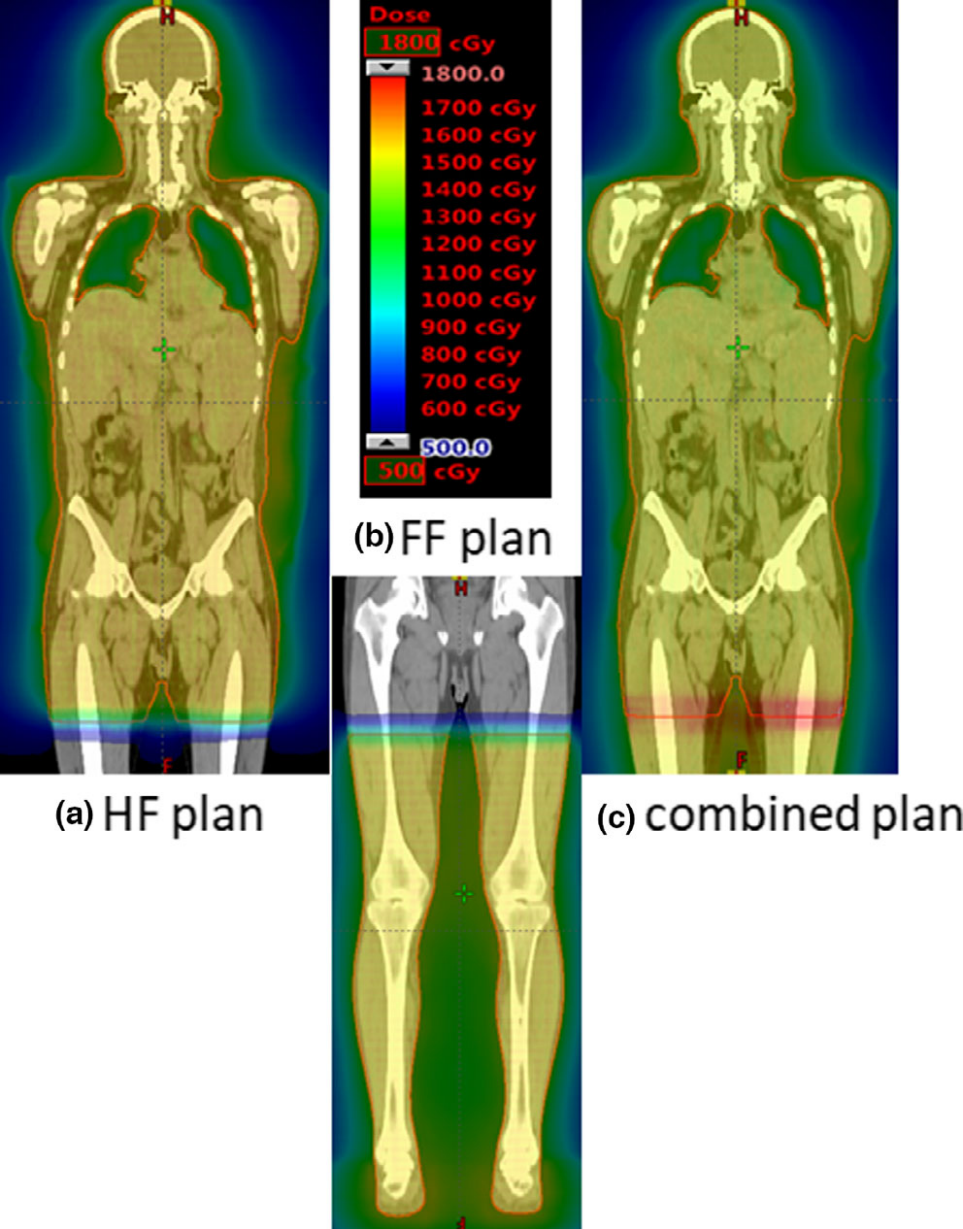
The intensity of the dose distribution at the junction between the HF and FF plans. The dose intensity at the junction between the HF and FF plans was attenuated in a gradation pattern. Parts a and b show the dose distributions of the HF and FF plans, respectively, and c shows the dose distribution of the combined plan.

The effects of rotational SE on the dose distribution were almost the same between the FF and HF plans. Areas of high‐ and low‐dose distribution were observed with increasing rotational SE. In the FF plan, statistically significant differences from the planned dose were observed at SE values of ≥1° for all indexes. Figure 5 shows the intensity of the dose distribution at the junction of the HF, FF, and combined plans. By narrowing the target volume at both body sides in the treatment planning, the dose intensity in the junction region was attenuated in a gradation pattern to ensure that its effect on the dose distribution at the confluence of the HF and FF plan images was kept within an acceptable range. Table 4 shows the results of the impact on the junction area in the overall combined plan. The CTVjt D_98%_ and D_95%_ values were not significantly different from the original planned dose at SE values of ≤4°. However, CTVjt D_2%_ was a statistically significant difference from the original planned dose at SE values of ≥2°.

**Table 4:** Effects caused by rotational setup error in the clinical target volume (CTV) junction area on the overall combined plan.

	Plan		Rotational SE (degrees) in pitch direction. (Average ± SD)				
			1.0	2.0	3.0	4.0	5.0
CTVjt	D_98%_ (Gy)	12.3 ± 0.3	12.6 ± 0.0	12.6 ± 0.0	12.4 ± 0.0	12.2 ± 0.1	11.5 ± 0.3*
	D_95%_ (Gy)	12.4 ± 0.3	12.7 ± 0.0	12.7 ± 0.0	12.6 ± 0.0	12.6 ± 0.0	12.4 ± 0.0
	D_2%_ (Gy)	17.4 ± 0.2	18.5 ± 1.1	20.5 ± 1.1*	21.0 ± 1.2*	20.9 ± 1.6*	20.5 ± 1.7*

* *P* < 0.05.

**Fig. 6:**
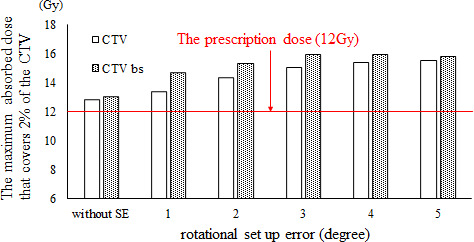
The near maximum dose to the clinical target volume (CTV) and body surface of the CTV (CTVbs) due to rotational setup error: change in D_2%_ (the minimum absorbed dose that covers 2% of the volume). The red line shows the prescribed dose of 12 Gy. The maximum dose for CTVbs without setup error was 13.1 Gy. With only 1° setup error, the near maximum dose increased to 14.7 Gy.

Specifically, the near maximum dose was increased at the CTVbs with increased rotational SE. Figure 6 shows the change of D_2%_ in the CTV and CTVbs at various values of rotational SE. In particular, the increase in the maximum dose to CTVbs that resulted from increased rotational SE was remarkable. The maximum dose to CTVbs without SE was 13.1 Gy, but the maximum dose with rotational SE of 1.0° increased to 14.7 Gy. Therefore, the maximum dose to the CTVbs increased by 12.7% when the rotational SE value was increased to 1°.

### 3.B Accumulated dose in the recalculation of delivered dose distribution

The residual of the rotational SE in the pitch direction, which cannot be corrected, was extracted for each patient. Table 5 shows an analysis of the angle of residual rotational SE in each focus region. The HF plan reflects the residual errors collated to the head, pelvis, and entire upper body, and the FF plan reflects those collated to the knee joint, ankle joint, and entire lower body. The maximum mean residual error in the HF plan was 1.0° for patient 4 with head collation, 2.3° for patient 8 with pelvic collation, and 0.6° for patient 8 with whole upper body collation. The maximum mean residual error in the FF plan was 2.1° for patient 2 with ankle joint collation, 1.5° for patient 6 with knee joint collation, and 1.0° for patient 2 with entire lower body collation.

**Table 5:** Angle of residual rotational setup error in each focus region. (degrees).

Correcting focused regions		Patients (Average ± SD)									
		1	2	3	4	5	6	7	8	9	10
Head first plan	Head	0.6 ± 0.7	0.3 ± 0.4	0.3 ± 0.2	1.0 ± 0.7	0.3 ± 0.2	0.6 ± 0.3	0.1 ± 0.2	0.4 ± 0.2	0.2 ± 0.0	0.4 ± 0.2
	Pelvis	0.9 ± 0.7	1.2 ± 0.4	0.6 ± 0.3	0.7 ± 0.4	0.7 ± 1.2	1.3 ± 0.4	0.4 ± 0.2	2.3 ± 0.5	0.4 ± 0.1	0.7 ± 0.2
	Whole upper body	0.2 ± 0.1	0.5 ± 0.3	0.4 ± 0.1	0.2 ± 0.2	0.4 ± 0.1	0.5 ± 0.1	0.1 ± 0.1	0.6 ± 0.1	0.1 ± 0.1	0.4 ± 0.1
Feet first plan	Ankle joint	0.3 ± 0.1	2.1 ± 0.2	1.1 ± 0.2	0.6 ± 0.6	0.6 ± 0.3	0.4 ± 0.4	0.5 ± 0.3	0.2 ± 0.2	0.4 ± 0.3	0.5 ± 0.3
	Knee joint	0.3 ± 0.1	0.8 ± 0.1	0.3 ± 0.1	0.5 ± 0.3	0.4 ± 0.2	1.5 ± 1.0	0.6 ± 0.3	0.7 ± 0.3	0.3 ± 0.1	0.4 ± 0.2
	Whole lower body	0.1 ± 0.1	1.0 ± 0.4	0.4 ± 0.2	0.4 ± 0.3	0.3 ± 0.1	0.2 ± 0.3	0.3 ± 0.2	0.1 ± 0.1	0.1 ± 0.1	0.4 ± 0.1

**Fig. 7:**
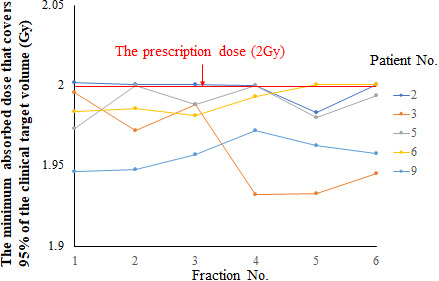
Actually delivered dose fluctuations per day in each of five patients. The daily prescribed dose was 2 Gy. Daily fluctuations were observed in the minimum absorbed dose that covers 95% of the clinical target volume.

**Fig. 8:**
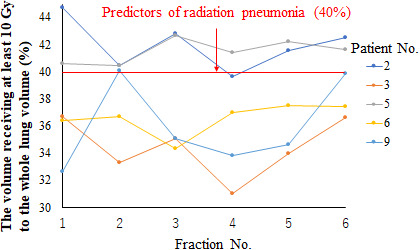
Fluctuations in actually delivered dose distribution to V_10Gy_ (the lung volume that receives at least a converted value of 10 Gy per day). Because the whole course was divided into six fractions, we performed V_10Gy_ conversion by multiplying the irradiation doses of each fraction by 6.

Figure 7 shows the fluctuations in recalculated delivered dose distribution results for D_95%_. The D_95%_ values in the CTV for each irradiation per day were a maximum of +0.1% and a minimum of −3.4%. Figure 8 shows fluctuations in the delivered dose distribution for the volume that receives a converted value of at least 10 Gy within the lungs throughout the entire course of treatment (10/6 Gy per day). The whole treatment course was divided into six fractions, therefore, each fractional irradiation dose was multiplied by 6 for performing V10 Gy conversion. The fluctuation of V_10Gy_ was larger than that of D_95%_, with a maximum of +11.9% and a minimum of −2.2%.

Table 6 compares the mean values of the 10 patients between the original planned dose distributions and the accumulated delivered dose distributions. Statistically significant differences were found between the original planned doses and delivered doses in V_110%_ of the CTV and D_2%_ of the CTVbs. The delivered doses increased by an average of approximately 1%. The other indicators were insignificantly different.

**Table 6:** Comparison between original planned dose distributions and delivered dose distributions.

Average ± SD		Planned dose distribution	Delivered dose distribution
CTV	D_98%_ (Gy)	11.7 ± 0.1	11.7 ± 0.1
	D_95%_ (Gy)	12.0 ± 0.0	12.0 ± 0.1
	D_2%_ (Gy)	12.9 ± 0.1	13.1 ± 0.1
	V_110%_ (%)	0.6 ± 0.1	1.6 ± 0.9*
	V_90%_ (%)	99.4 ± 0.4	99.6 ± 0.2
Lung	Mean (Gy)	9.5 ± 0.1	9.5 ± 0.3
	V_10 Gy_ (%)	38.4 ± 1.7	37.8 ± 5.4
CTVbs	D_98%_ (Gy)	11.0 ± 0.4	10.5 ± 0.3
	D_2%_ (Gy)	13.1 ± 0.1	14.0 ± 0.6*
CTVls	D_98%_ (Gy)	11.1 ± 0.2	10.8 ± 0.4
	D_2%_ (Gy)	12.8 ± 0.1	12.8 ± 0.1

Delivered dose distributions mean the integration of the dose distributions for each actual irradiation.

* *P* < 0.05.

## 4 DISCUSSION

TBI using HT is a promising treatment modality. However, it is necessary to improve the treatment’s accuracy. In this study, the permissible range of uncorrectable rotational SE in the pitch direction was evaluated with a focus on the impact on the irradiated patient dose distribution.

Larger SE in the pitch rotation direction is associated with greater dose inhomogeneity in the CTV. For the 10 patients, when the SE value in the pitch direction was 3°, the mean ± SD (%) of V_110%_ and V_90%_ of the CTV in the HF plan were 7.6% ± 3.4% and 98.6% ± 0.2%, respectively (Table 2). Therefore, the dose distribution error of CTV was acceptable at rotational SE values of ≤3°. Conversely, the mean value of V_110%_ in the CTV exceeded the tolerable deviation of 10% when the SE value was 4°. Dose heterogeneity needs to be kept within ±10% of the prescription dose ^14^, so the dose heterogeneity caused by rotational SE of ≥4° is unacceptable. Moreover, the lung dose results showed that V_10 Gy_ was kept under 40% when the SE value was ≤2°. These simulation results indicate that dose errors due to SE of <2° are acceptable. Similarly, for the 10 patients, when the rotational SE was 2°, the mean ± SD (%) of V_110%_ and V_90%_ of the CTV in the FF plan were 5.3% ± 2.4% and 99.6% ± 0.1%, respectively (Table 3).

The results of the impact on the junction area in the overall combination plan showed that CTVjt D_2%_ was a statistically significant differences from the original planned dose at SE values of ≥2°. When the rotational SE was 2°, the near maximum dose in the CTVjt was 18% higher than that without SE. However, D_98%_ and D_95%_ were not significantly different from the original planned dose at SE values of ≤4°. In addition, the average value of V_110%_ in whole CTV was ≤10%, therefore, because the dose increased only in the local region at the rotational SE value of 2°, the effect on the entire CTV was small.

The simulation results showed that a high‐dose region was caused by the SE, with D_2%_ and V_110%_ showing significant increases even at the rotational SE value of 1°. Specifically, the maximum dose in the CTVbs increased with increased rotational SE. Figure 6 shows that the effects on the CTVbs value were quite large: the D_2%_ value was increased by 12.7% at the SE value of only 1°. There is a high‐beamlet fluence area around the body surface to maintain a sufficient dose in the build‐up region.^10^ Therefore, the rotational SE increases the radiation dose at the patient’s body surface. The results of the FF plan showed larger dose deviations than those of the HF plan. Because the contour of the foot shape was more complex because of structures such as the toes and heel, these beamlet fluences were associated with steep distributions. Therefore, smaller SEs induced larger dose distribution errors in the FF plan.

The dose distribution results in clinical cases (Section 3.B) showed that the D_95%_ of the CTV was not significantly different from the planned dose distribution based on the original images. However, the high‐dose region represented by V_110%_ increased by approximately 1.0%. The results in the clinical cases are consistent with the simulation results shown in Section 3.A.

When the actual irradiation dose was calculated, as shown in Fig. 7, the D_95%_ of CTV fluctuated daily, and the minimum value was −3.4%. However, the cumulative dose distributions did not show a significant difference from the planned dose distributions. We consider that this is because the part where the dose changes because of daily SE is accidental, and in the case of a CTV with large volume, the parts where the dose increases and decreases with respect to the planning dose cancel each other out. In contrast, the dose to the lungs varied greatly from day to day compared with that to the CTV, increasing by up to about 12%. Because of the anatomical structure of the lungs, they are surrounded by steep dose‐increasing areas in all directions. Therefore, we believe that the dose increase was caused by the lungs’ movement into the target area as a result of the rotational SE.

In this study, the uncertainty of the DIR process for dose accumulation with the MVCT dose distributions was not taken into consideration. However, the impact of this error is limited because body and lung contours were used as landmarks during DIR, and these regions have large differences in pixel values from the surrounding areas.

A previous study reported that systemic SE gradually increased from cranial to caudal in craniospinal irradiation using HT.^16^ However, the application of IGRT with MVCT can improve the uncertainty of SE values.^19^ Therefore, acquiring images of not only a specific area but also the whole‐body region is practical in TBI using HT. Regarding methods of correcting rotational SE, an algorithm that corrects the rotational SE value in HT using computerized translational bed movement^20^ and a device that corrects error by being attached to a treatment table^21^ have been reported but not commercialized.

In this study, we conducted simulations that included the rotational SE derived from both the patient’s own position and the sagging of the treatment couch. This couch sagging depends on the patient’s weight. However, we could not apply a weight dependence factor to this SE error value. We evaluated the acceptable level of this rotational SE value in the dose distribution. Currently, the development of new HT systems is making improvements focused on this problem.^22^ A so‐called couch catcher, which prevents the couch from sagging by supports the superior end of the couch, has been added. The newest release of the HT system may thus suppress systematic rotational SE at the level of the device. However, complete elimination of the rotational SE will be impossible because it is also caused by the patient’s posture.

This study also included cases in which repositioning and re‐IGRT were performed manually during the IGRT process. When performing IGRT, it was necessary to decide whether to continue or interrupt the treatment and manually correct the position, and the time spent on re‐IGRT was a problem. The MVCT image data collection time is 5 seconds per slice, and required for an additional 16 seconds. So the total scan time is 5 seconds / slice + 16 seconds. In addition, HT‐TBI requires changes in the patient’s position (HF plan and FF plan), so the total treatment time of TBI with HT is longer than that of general local radiation therapy. However, in IGRT, SE correction results in a tradeoff between extended treatment time and improved reproducibility. Therefore, a rationalization for SE correction procedures in IGRT is beneficial because it can avoid unnecessary extension of treatment time. Establishing an acceptable SE value by quantitatively evaluating the effects of pseudo‐rotational SE simulation on the dose distribution can rationalize the SE correction procedure.

In this study, rotational SE was found to cause an unexpected expansion of the high‐dose region near the contour surface of the patient’s body. Therefore, a future task is to improve treatment planning methods to reduce the effects of rotational SE on the dose distribution.

## 5 CONCLUSION

The effect of rotational SE values of ≤3° on dose heterogeneity in the CTV was within ± 10% of the prescribed dose. We conclude that the rotational SE value range of ≤2° is an acceptable limit. This is because the results in the high‐dose region of the lung became unacceptable when the rotational SE was ≥3°. By clarifying the permissible value of rotational SE, it is expected that the SE correction procedure in IGRT will be rationalized and that burden on the patient due to the extension of treatment time will be prevented. However, rotational SE in TBI treatment using HT may increase the radiation dose, particularly on the patient’s body surface.

## AUTHOR CONTRIBUTIONS

Akira Isobe: Conceptualization, Methodology, Formal analysis, Visualization, Writing—Original Draft; Keisuke Usui: Methodology, Project administration, Supervision, Writing—Review and Editing; Naoya Hara: Validation; Keisuke Sasai: Writing—Review and Editing.

## CONFLICT OF INTEREST

The authors declare that they have no conflict of interest.

## ETHICAL APPROVALT

Since this study was a noninterventional and noninvasive study, informed consent is omitted. Instead, posting information about the conduct of the study, including the purpose of the study, on the website of the Department of Radiology, Juntendo Hospital, guarantees the opportunity for the subject to reject.

This study was approved by the institutional review board of our hospital (reception number: 18‐034 and 20‐105). All procedures performed in studies involving human participants were in accordance with the ethical standards of the Institutional Review Board (IRB) and with the 1964 Helsinki declaration and its later amendments or comparable ethical standards.

## Data Availability

The data that support the findings of this study are available on request from the corresponding author. The data are not publicly available due to privacy or ethical restrictions.
